# Judgments of Sexual Attractiveness: A Study of the Yali Tribe in Papua

**DOI:** 10.1007/s10508-012-9906-x

**Published:** 2012-02-14

**Authors:** Piotr Sorokowski, Agnieszka Sorokowska

**Affiliations:** Institute of Psychology, University of Wroclaw, ul. Dawida 1, 50-527 Wroclaw, Poland

**Keywords:** Physical attractiveness, Waist-to-hip ratio (WHR), Sexual dimorphism in stature (SDS), Leg-to-body ratio (LBR), Pig ownership, Yali (Papua)

## Abstract

Preferences for waist-to-hip ratio (WHR), sexual dimorphism in stature (SDS), and leg-to-body ratio (LBR) have been investigated predominantly in Western cultures. The aim of the present study was to examine the preferences of a relatively isolated, indigenous population (i.e., Yali of Papua, inhabiting the mountainous terrain east of the Baliem valley). A total of 53 women and 52 men participated in the study. Study sites differed in distance from Wamena, the biggest settlement in the region, and frequency of tourists’ visits. We found that the mate preferences among Yali men and women for WHR, LBR, and SDS were not exactly the same as in Western samples. Yali preferred low women’s WHR and relatively high women’s (but not men’s) LBR. Women’s and men’s ratings of each SDS set were similar, which suggests that the “male-taller norm” in Yali tribe was far weaker than in Western cultures. Additionally, the observed preferences were modified by contact with different cultures, age, and accessibility of food resources (pig possession). Our results suggest that human norms of attractiveness are malleable and can change with exposure to different environments and conditions.

## Introduction

The importance of physical attractiveness for mate selection has been known since the Walster, Aronson, Abrahams, and Rottman (1966) study. They investigated factors that influence an interest for a potential partner during “blind dates” (e.g., masculinity/femininity, personality, intelligence, similarity). The most important characteristic was found to be physical attractiveness. Further studies have shown that, in mate selection, physical attractiveness seems to be universally important. It was shown, for example, by Buss (1989) in his cross-cultural research conducted in 37 cultures. Lippa (2007) replicated that finding in 53 countries (internet study on 200,000 people).

For several decades, psychologists have been studying ideals and elements of beauty. After 1966, the term “physical attractiveness” was used in more than 1700 articles in WEB of Science (ISI Web of Knowledge) and Google Scholar links this topic with 357,000 works. However, it should be highlighted that the majority of these studies were conducted among Western societies (or industrialized Asian countries like Japan or China). The low cultural variation of the samples is a weakness, limiting the possibility of investigating the universality of the human attractiveness or generalizing the findings (e.g., Cunningham, Roberts, Wu, Barbee, & Druen, 1995; Langlois et al., 2000).

Data from cultures weakly influenced by Western cultures are scarce (e.g., the Matsigenka of Peru: Yu & Shepard, 1998; the Hadza of Tanzania: Wetsman & Marlowe, 1999; Bakossi of Cameroon: Dixson, Dixson, Morgan, & Anderson, 2007; Shiwiar of Ecuador: Sugiyama, 2004; Kanite of Papua New Guinea: Dixson, Sagata, Linklater, & Dixson, 2010). These studies show, however, that preferences for body shape are not identical across the world. Because in different populations (e.g., hunter-gatherers vs. highly developed) various phenotypic traits can be adaptive, classical natural and sexual selection theories might explain the existence of these differences. For example, one of the biological mechanisms proposed on the basis of natural and sexual selection and explaining the existing cross-cultural differences in attractiveness preferences is the central tendency (Symons, 1979), i.e., preference for the average for particular traits. Symons suggested that the average in the local population often approximates the naturally selected optimal design and this presumption was confirmed by numerous studies (e.g., Jones, 1996; Jones & Hill, 1993).

Which elements of the human body determine its attractiveness? The results of research have linked it with, among others, waist-to-hip ratio (WHR), height or sexual dimorphism in stature (SDS), and leg-to-body ratio (LBR). A short review concerning preferences for these body proportions is presented below.

### Waist to Hip Ratio Preferences

Singh (1993) showed that WHR close to 0.7 is one of the most important markers of female attractiveness. Singh suggested that WHR is a reliable marker of reproductive abilities and women’s health. Many studies conducted both before and after Singh’s observation confirmed his hypothesis. First of all, WHR (distribution of adipose tissue) is a result of the activity of male and female sex hormones (Björntorp, 1991; Jasieńska, Ziomkiewicz, Ellison, Lipson, & Thune, 2004). Low WHR is also a reliable marker of probability of conception during in vitro fertilization (Waas, Waldenstrom, Rossner, & Hellberg, 1997). Additionally, the WHR of women decreases (becomes more attractive) during puberty and increases again after menopause (Kirschner & Samojlik, 1991). Finally, it was shown that low WHR is a reliable marker of a woman’s health (e.g., cardiovascular diseases, diabetes) (Singh, 2006).

Probably because of the relationship between reproductive health and WHR, this trait was suggested to be a relatively universal attractiveness marker. Similar results to the classical Singh (1993) study were obtained in, among others, Europe (e.g., Furnham, Tan, & McManus, 1997; Rozmus-Wrzesinska & Pawlowski, 2005), Indonesia (Singh & Luis, 1995), Guinea Bissau (Singh, 2004), Cameroon (Dixson et al., 2007), and Papua New Guinea (Dixson, Sagata et al., 2010). Therefore, it can be presumed that men’s attraction to women of certain body proportions might indicate a natural and universal preference for healthy and fertile partners.

Data obtained in a few hunter-gatherer societies, like the Hadza tribe from Tanzania, the Matsiguenka from Peru or the Shiwiar from Ecuador, showed that high WHR was preferred in women in such populations (Marlowe & Wetsman, 2001; Sugiyama, 2004; Wetsman & Marlowe, 1999; Yu & Shepard, 1998). A few hypotheses explaining such preferences exist, but generally it was suggested that it was a result of the stimuli used. In a recent study, Marlowe, Apicella, and Reed (2005) showed line drawings that varied in WHR with a profile view of a silhouette designed to look like a young Hadza woman whose buttocks were visible. In this study, Hadza men judged the 0.6 WHR to be most attractive.

Additionally, in non-industrialized populations, women of higher body mass are preferred (Cassidy, 1991) and silhouettes of higher WHR (when WHR is modified by changes in width of waist) are perceived as having higher body mass index (BMI) (Rozmus-Wrzesińska & Pawlowski, 2005). The question of whether female WHR is equally as important as Body Mass Index (BMI) in attractiveness ratings is currently under debate. On the one hand, some researchers suggest it is the BMI that drives mate selection, as it accounts for greater variance in attractiveness scores than WHR (Tovee, Maisey, Emery, & Cornelissen, 1999). On the other hand, many new studies have confirmed the high importance of WHR in perceiving female silhouettes. It has been shown that in both Western (Singh & Randall, 2007) and non-Western cultures (Dixson, Li, & Dixson, 2010; Singh, Dixson, Jessop, Morgan, & Dixson, 2010) women who had undergone micrograft surgery that decreased their WHR were judged to be most attractive regardless of fluctuations in their BMI. Further, Platek and Singh (2010) have shown, using fMRI brain scanning, that men’s brain areas concerned with reward showed significantly greater activation when they viewed post-operative, as compared to pre-operative, micrograft images.

### Height Preferences

Height is regarded as one of the most important characteristics of men’s physical attractiveness and may thus serve as an initial criterion for women to decide upon further interest and engagement in a courtship situation (Pierce, 1996). Previous research in Western societies has shown that women prefer relatively tall men as potential partners whereas men prefer women slightly shorter than themselves (Pawlowski & Koziel, 2002; Salska et al., 2008; Shepperd & Strathman, 1989). Recent research has emphasized the significance of relative (rather than absolute) body height when studying men’s and women’s height preferences in a romantic relationship. Such research involves investigation of preferences for the difference between one’s own height and the height of a preferred partner (Fink, Neave, Brewer, & Pawlowski, 2007; Pawlowski, 2003). Taken together, these studies showed that both men and women adjusted their SDS preferences according to their own body height; however, a few participants chose the option of a woman being slightly taller than a man.

To date, ET LEAST two studies have reported data that question the universality of the “male-taller norm.” Sear and Marlowe (2009) reported that in the Hadza society (Tanzania), in 8.2% of 207 marriages the wife was taller than the husband, which was significantly higher than in Western societies. Sorokowski, Sorokowska, Fink, and Mberira (2012) reported data on SDS preferences of another traditional ethnic group—the Himba of northern Namibia. Contrary to Western societies, many Himba preferred partners of height equal to their own. This finding demonstrates that the “male-taller norm” reported in Western samples, was much less pronounced in the Himba tribe. It is known that people’s variation in body height is affected not only by genetic differences, but also by environmental influences. Lower height in humans can result from malnutrition, stress or various infectious diseases (Beard & Blaser, 2002). This is why it might be hypothesized that height preferences may be influenced by environmental and ecological conditions (e.g., in harsh conditions taller height might be preferred).

Height, similarly to WHR, might also be related to the reproductive success of an individual. Within Western populations, taller men had greater reproductive success (e.g., U.S.: Mueller & Mazur, 2001; Poland: Pawlowski, Dunbar, & Lipowicz, 2000) and the opposite relationship was observed in women (e.g., UK: Nettle, 2002). However, the results from populations of natural birth control are less consistent. For example, some studies showed the opposite correlation between male height and number of children (e.g., !Kung San from Northern Namibia: Kirchengast & Winkler, 1995; broad description of research about height and reproductive success: Sear, 2010).

### Leg Length Preferences

Another morphological feature possibly influencing the judgments of attractiveness is the leg-to-body ratio (LBR). Several reasons why leg length could have an impact on the general human attractiveness can be indicated: (1) Relative leg length might be a credible cue to health status (Davey Smith et al., 2001; Lawlor, Taylor, Davey Smith, Gunnell, & Ebrahim, 2004) and the early childhood environmental influences on the organism (illnesses, malnutrition) (Wadsworth, Hardy, Paul, Marshall, & Cole, 2002); (2) Relatively short legs in women might be a sign of lower reproductive capabilities (Fielding et al., 2008); (3) Leg length might be an indicator of biomechanical efficacy (e.g., running or swimming ability) (Cavanagh & Kram, 1989), which was important in our evolutionary past. For a review and broader explanation of these parameters see Bogin and Varela-Silva (2010).

It was shown that people perceive a relatively high (but not extremely high) LBR as attractive in women (Bertamini & Bennett, 2009; Rilling, Kaufman, Smith, Patel, & Worthman, 2009; Sorokowski & Pawlowski, 2008; Swami, Einon, & Furnham, 2006; but see Frederick, Hadji-Michael, Furnham, & Swami, 2010) and men (Sorokowski & Pawlowski, 2008, Sorokowski et al., 2011; but see Swami et al., 2006). At the same time, excessively long or short legs were perceived as less attractive in both sexes. This is consistent with the notion that large deviations of any trait from the population average are usually maladaptive and therefore perceived as less attractive (Symons, 1979).

Data regarding LBR preferences in different cultures were collected by Sorokowski et al. (2011), who investigated it in 27 nations. While the silhouettes with short and excessively long legs were perceived as less attractive and silhouettes with LBRs close to the average were perceived as most attractive across all nations, too long legs were generally more attractive than too short. The LBR preferences were only slightly modified by the participants’ origin. Europeans, together with Canadians and Africans, preferred a relatively high LBR, and Latin Americans a relatively low LBR. However, the majority of participants originated from urban areas within their respective countries—still, the preferences for high LBR seemed not to be directly related to contact with Western culture (as Nigerians and Georgians preferred higher LBR than Britons and Canadians).

Studies demonstrating relatively high cross-cultural differences in LBR preferences also exist. Swami, Einon, and Furnham (2007) showed that, for British participants, a relatively high LBR was preferred in women and a relatively low LBR in men, whereas rural Malaysian participants rated medium female LBR and low male LBR as the most attractive. Also, the results obtained among the Himba people (Sorokowski, Sorokowska, & Mberira, in press) differed from previous studies (Bertamini & Bennett, 2009; Sorokowski & Pawlowski, 2008; Sorokowski et al., 2011; Swami et al., 2006). It was the first population where attractive female LBR was lower than attractive male LBR. These results showed that leg length might not be a universal attractiveness marker and preferences for this body component could be related to a specific environment or culture.

As preferences for described body proportions (WHR, SDS, LBR) were investigated mainly in Western cultures, the aim of the current study was to examine the preferences of a relatively isolated population (i.e., the Yali of Papua).

## Method

### Participants

The research was conducted among the Yali tribe (Papua, Indonesian province, previously known as West Papua or Irian Jaya). The Yali inhabit the mountainous terrain east of the Baliem valley (central part of Papua). Because of difficult accessibility, majority of these terrains had not been explored until about 50 years ago (Koch, 1974). Yali society is traditional and strictly male-dominated. Part of the Yali population still preserves traditional clothing style-men wear only a phallocrypt (*koteka*, a decorative penis sheath) and women a short grass skirt.

A total of 105 people (53 women, 52 men) participated in the study. The women were aged between 20 and 50 years (*M* = 34.8, *SD* = 7.6), and the men between 25 and 60 years old (*M* = 36.4, *SD* = 8.7). The age of each participant was self-estimated. Yali people have a longitudinal concept of time—the question about age in “Western years” was absolutely understandable for them. However, the majority of participants did not know exactly how old they were and were giving an approximate age. Participants received a fee of about 2 USD for their participation in a series of similar studies.

The study was conducted north of the summit of Mount Elit (also known as Gunung Elit, about 4000 m). All the study sites were located along a mountainous route (from South-East to North-West) surrounding the Baliem Valley from the East (hand-made map available upon request from the corresponding author). The first, southernmost village, relatively often visited by tourists (10–15 trek groups yearly) was Piliam (*n* = 36). Other, relatively isolated mountain villages (listed from the closest to the farthest from Piliam) were: Pui (*n* = 6), Hiklahin (*n* = 33), Ohomtongo (*n* = 8), Sali (*n* = 2), Fik-Fak (*n* = 8), Mogobie (*n* = 3), Penyam (*n* = 3), and a few other small settlements where single families lived (*n* = 6). As was indicated by participants themselves, the more isolated villages were visited by trekking groups around 1–3 times in the last 5 years. Names of all villages are written in local spelling. We were not able to compare all of them with the names on the official maps of the region (they are hardly available).

In all the studies, we controlled for following variables: time from last encounter with tourists (in months), distance from Wamena (in days’ walk), and participants’ wealth (number of possessed pigs).

The villages where the study was conducted were located in the mountains a few dozen kilometers north-east of Wamena, a central part of Papua. It is the biggest settlement (around 10,000 people) in the Baliem Valley. The only possibility to access the Baliem Valley and nearby mountains is a local flight to Wamena. Therefore, it is a political, touristic and trade center of this region. Although the study sites were located within similar absolute distance from Wamena, their actual contact with tourists, and Indonesian people depends on convenience of the communication trail. According to Indonesian government policy, increasing numbers of Indonesians (traders, clerks, officials, soldiers, policemen, etc.) settle down in Wamena. Additionally, all tourist treks in the Baliem Valley start and finish in Wamena and majority of tourists do not reach villages located more than 2–3 days walk from the city.

On the other hand, the Yali people (and other tribes) have maintained regular trading links with the Dani of the Baliem Valley, supplying them with items which are no longer available in the valley because of deforestation. Salt, a highly prized commodity, was often traded in return (Matthiessen, 1989). Presently, Yali also buy things available in Wamena shops, like tools, etc. Additionally, some Yali villages have contact with other cultures via doctors, “mobile” nurses traveling around the area, or Christian missionaries present in the region. In summary, a greater walking distance from Wamena means more isolation from other (Indonesian, Western) cultures.

Another variable describing participants’ isolation was time since last encounter with tourists. Because of terrain formation, size of a village, accessibility and attractiveness of route, different villages differ in frequencies of tourist visits. Although some northern villages are within relatively short walking distance from the Baliem Valley, they are virtually never visited by tourists.

A variable indicating participants’ wealth was their number of pigs. Many previous studies have shown that, in the indigenous cultures of New Guinea, pigs carry significant social and cultural as well as financial value (Boyd, 1985; Minnegal & Dwyer, 1997). Pigs are the most important currency in the west Papuan highlands region and possessing pigs is a reliable marker of Yali wealth (e.g., wives are bought with pigs). Additionally, the number of possessed pigs is probably a good indicator of Yali health, which is probably related to better nutrition of wealthier families (e.g., we found that number of pigs positively correlated with number of children; *r* = .38, df = 104, *p* < .001).

### Procedure

As the study was a novelty for the local people, we did not simply show them the stimuli. Before the study, the participants were told that the study involved judging what they find attractive in the opposite sex and what they do not. We explained in detail what each picture depicted and ensured that the participants understood the procedure (e.g., when examining SDS preferences, we asked first if they prefer tall or short partners and after showing the stimuli, we made sure they chose a picture depicting their declaration). We presented three different sets of stimuli:

### Measures

#### Waist-to-Hip Ratio

**Fig. 1:**
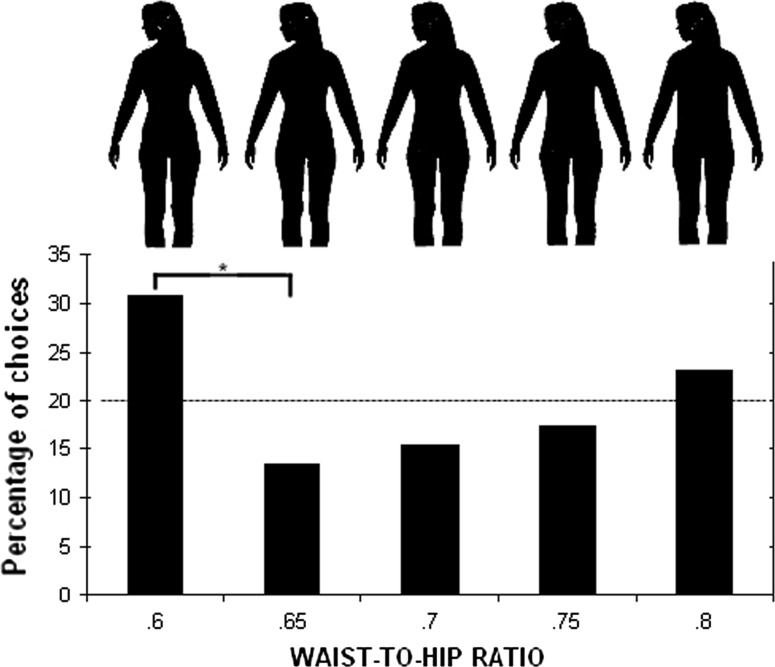
Preferences for women’s WHR in Yali tribe

As stimuli, five female blackened silhouettes with five different WHRs (.60, .65, .70, .75, .80) were presented. We modified (blackened) the pictures taken from the Rozmus-Wrzesinska and Pawlowski (2005) study (see Fig. 1). The silhouettes were not based on WHR measurements in the Yali population, because the measurement would have been too invasive in this culture (what is more, some older Yali women wore only a short, protruding grass skirt, which obstructed estimation of WHR and it was impossible to ask them to take it off). The Yali men were asked to indicate the most attractive silhouette from the given set.

#### Sexual Dimorphism in Stature

The participants were shown six pairs of silhouettes, depicting an opposite sex couple differing in SDS, calculated as the ratio of the man’s height divided by the woman’s height. The SDS ratios ranged from 1.19 (i.e., the man being much taller than the woman) to 0.96 (i.e., the woman being slightly taller than the man) (for details on the material, see Pawlowski, 2003). Participants were asked to choose the pair they would prefer for their own relationship. Our SDS stimuli were adequate for the Yali population because their real SDS is similar to that in Western societies (women: *M* = 142.9 cm; *SD* = 4.25, *n* = 52; men: *M* = 151.0 cm; *SD* = 6.9; *n* = 53; SDS: 1.07).

#### Leg-to-Body Ratio

As stimuli, five male and five female blackened silhouettes were presented (for details on the material, see Sorokowski & Pawlowski, 2008). We changed the original stimuli according to Yali LBR measurements (men’s LBR: *M* = .449, *SD* = .02; women’s LBR: *M* = .445, *SD* = .014, measured as sitting height/height for 30 men and 30 women). We used small 5% steps between figures to obtain five male and five female stimuli of different LBRs (the average picture LBR .45, pictures with elongated legs LBR .475, and LBR .5, and pictures with shortened legs LBR .425, and LBR .40) (see Figs. 3; 4). The participants were asked to choose the most attractive silhouette. Female silhouettes were rated only by men and male silhouettes were rated only by women.

## Results

The obtained percentages were compared with the percentage expected by chance (equal distribution of responses across the 6 categories) using a χ^2^ test. To investigate the differences between percentages, a test comparing two proportions was used (a two-sided test for analyses without directional hypotheses).

### Men’s Preferences for WHR

More than 30% of men rated the female image depicting a WHR .60 as the most sexually attractive (Fig. 1), which was significantly more as compared to WHR .65 (*p* < .05). The WHR .80 image was the second most favored choice, but the percentage of choices did not differ from expected levels or from percentages of choices of the other WHRs (Fig. 1).

### Women’s and Men’s Preferences for SDS

**Fig. 2:**
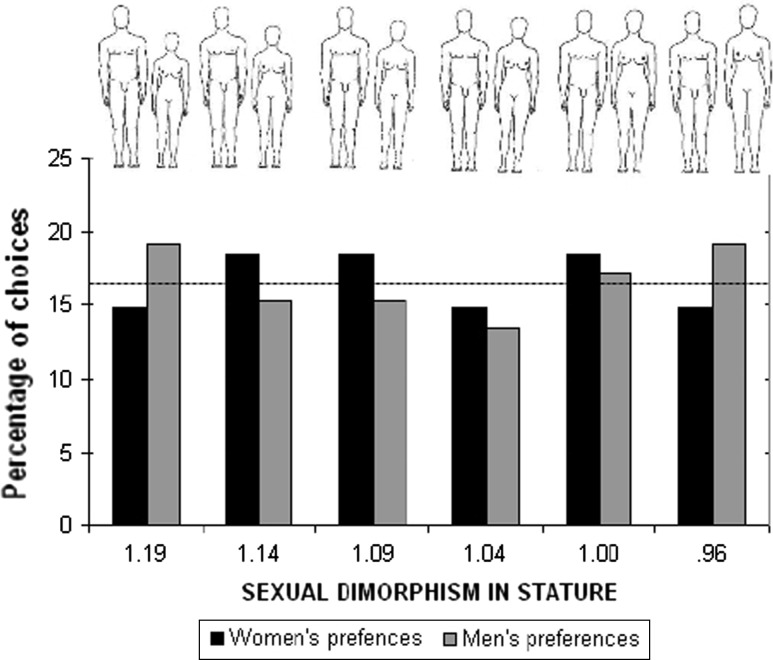
Preferences for SDS in Yali tribe

The number of participants expressing preferences for any of the SDS sets did not differ from the levels expected by chance as revealed by χ^2^ tests both for the men’s choices (χ^2^ = 1.2, df = 5) and the women’s choices (χ^2^ = 1.7, df = 5) (Fig. 2).

### Women’s and Men’s Preferences for LBR

**Fig. 3:**
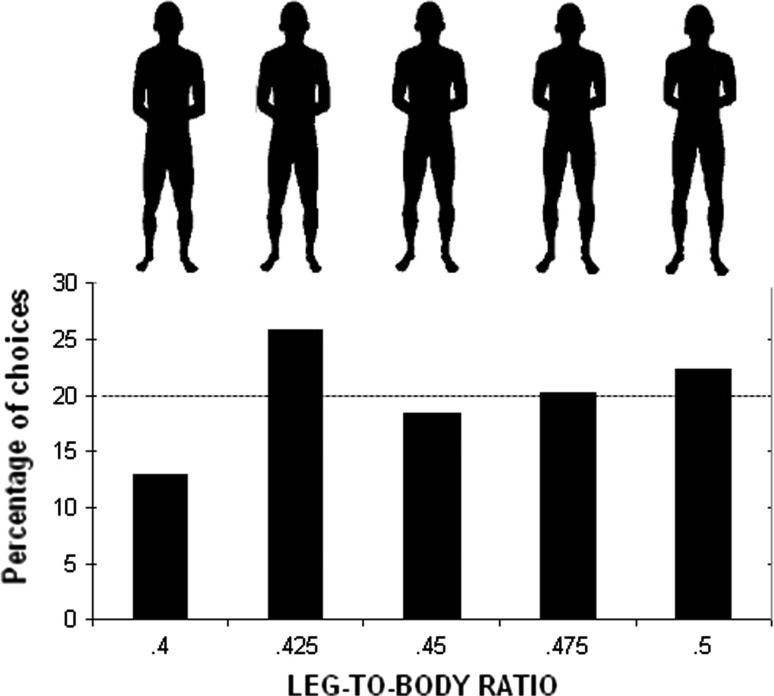
Preferences for men’s LBR in Yali tribe

**Fig. 4:**
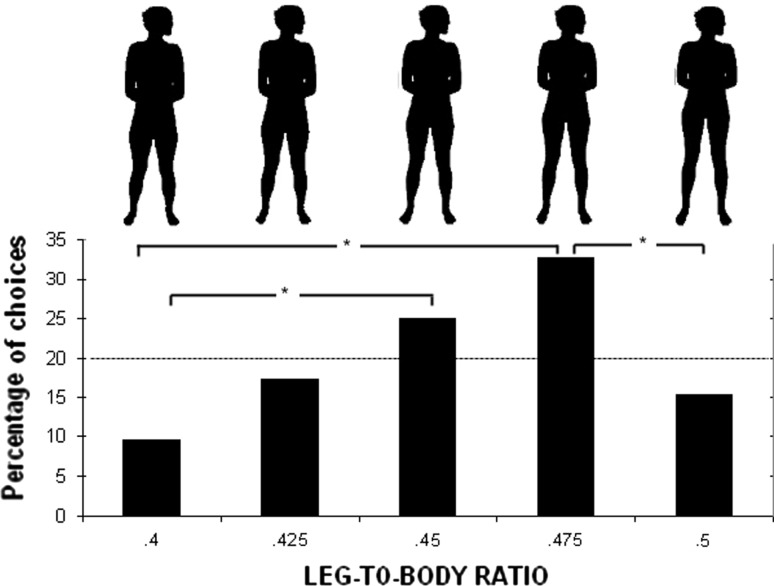
Preferences for women’s LBR in Yali tribe

The numbers of women expressing a preference for any of the LBRs did not differ from the levels expected by chance as revealed by χ^2^ tests for the women’s choices (χ^2^ = 4.6, df = 4) (Fig. 3). Men’s choices differed from the levels expected by chance (χ^2^ = 16.1, df = 4, *p* = .003). Almost 33% of the men rated the female image depicting the LBR .475 as the most sexually attractive (Fig. 4), which was significantly more than LBR .4 and LBR .5 (*p*s < .05). The .45 LBR image was the second most favored choice (Fig. 4) and was preferred significantly to LBR .4 (*p* < .05).

### Correlations between Preferences and Distance from Wamena, Last Encounter with Tourists, Number of Possessed Pigs, and Participants’ Age

All correlations are shown in Table 1. We found that Yali men who lived farther from Wamena preferred taller women of higher WHR. Moreover, Yali men and women who had not seen any tourists for a long time and Yali women who lived farther from Wamena preferred relatively long-legged silhouettes. We also found that Yali men who possessed fewer pigs preferred higher LBR (at the trend level) and WHR. Men’s age correlated with preferred SDS in a relationship—older Yali chose a relationship with taller women.

**Table 1:** Correlations between preferences and distance from Wamena, last encounter with tourists, number of possessed pigs, and participants’ age

	Sex of participant	*N*	df	Distance from Wamena (in days’ walk)	Last encounter with tourists (in months)	Number of possessed pigs	Age
Sexual dimorphism in stature	Female	53	51	−.14	.01	.08	−.17
	Male	52	50	.30**	.18	.00	.46***
Leg-to-body ratio	Female	53	51	.30**	.26*	.15	.08
	Male	52	50	−.13	.27**	−.30**	.04
Waist-to-hip ratio	Male	52	50	.30**	.18	−.26*	.02

* *p* < .10; ** *p* < .05; *** *p* < .01

### Differences in Preferences Between Inhabitants of Piliam and Inhabitants of More Remote Villages

As Piliam is the closest to Wamena and the most frequently visited by tourists, we compared the results obtained in this village with other, more remote villages. To test the effect of region and sex on participants’ preferences, analysis of covariance (ANCOVA*)* was used. Design of this study was a 2 (Piliam vs. other villages) × 2 (participant’s sex), controlling for participants’ age and number of pigs (covariates).

**Fig. 5:**
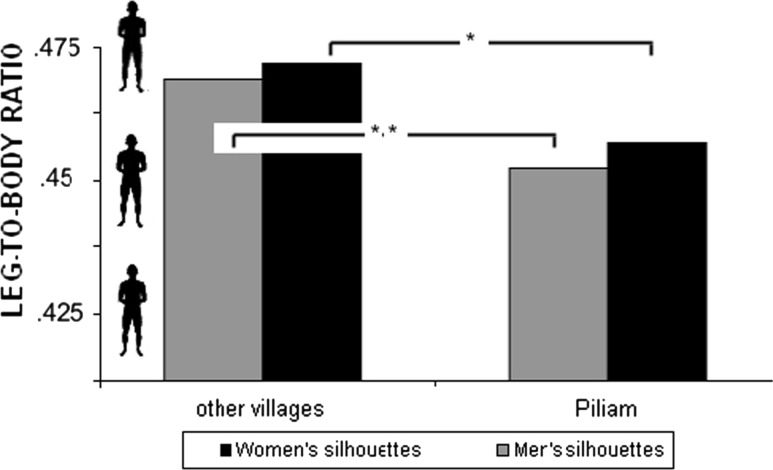
Preferences for LBR in Piliam and other villages

In the case of SDS, neither main effect of sex, *F*(1, 100) = 1.2, nor main effect of region, *F*(1, 100) = 3.1, were significant predictors of participants preferences for partner’s height. Also the interaction effect “sex” × “region” was non-significant, *F*(1, 100) = 1.5; however, LSD (least significant difference) post hoc test, showed that men who lived in Piliam (*M* = 1.1, *SD* = .07) preferred significantly higher SDS (i.e., shorter women) than men living in more remote villages (*M* = 1.05, *SD* = .07), *p* < .05 (see Fig. 5).

**Fig. 6:**
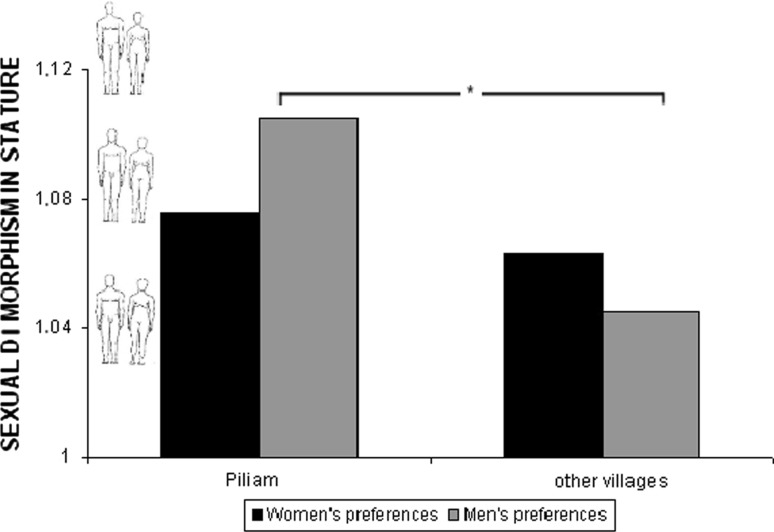
Preferences for SDS in Piliam and other villages

In the case of LBR, main effect of sex, *F*(1, 100) < 1, was a non-significant predictor of participants’ LBR preferences. However, main effect of region, *F*(1, 100) = 6.9, *p* < .01, *η*_p_^*2*^ = .06, was significant: participants from more remote villages preferred longer legged silhouettes. Despite a non-significant interaction effect, *F*(1, 100) < 1, LSD post hoc test showed that women living in more remote villages (*M* = .45, *SD* = .03) preferred longer legged men than women living in Piliam (*M* = .43, *SD* = .03) (*p* < .05) and men living in more remotes villages (*M* = .46, *SD* = .04) preferred longer legged women more than men living in Piliam (*M* = .44, *SD* = .03) (*p* = .06) (Fig. 6).

The analyses did not confirm the effect of region on WHR preferences in men, *F*(1, 48) < 1.

## Discussion

Our results present preferences for body proportions in the Yali population. The study was conducted in the population living in one of the last parts of the world which is relatively isolated from Western culture. Some results obtained in the Yalimo region differed from preferences observed in previous studies regarding attractiveness. Additionally, our results suggest that the aesthetic preferences are malleable and can change with exposure to different environments and conditions.

Women’s and men’s ratings of each SDS set were similar. It suggests that the participants either did not have specific SDS preferences (and their choices were random) or that they were very diverse and were equally divided between each presented SDS. In any case, this findings differs from several dozen previous studies showing strong “male-taller norm” (Fink et al., 2007; Pawlowski, 2003; Pawlowski & Koziel, 2002; Pierce, 1996; Salska et al., 2008; Shepperd & Strathman, 1989). In the Yali tribe, the “male-taller norm” was far weaker than in Western cultures. More than 60% of participants preferred relationships where a man was taller than a woman; however, it was significantly lower than in Western societies (i.e., 96.3% in Poland, Pawlowski, 2003). Additionally, Yali men who were older and lived farther from Wamena preferred taller women, which suggests that male preferences for female height may change and that 10 or 20 years ago men may have had a greater preference for tall women than was observed among present day Yali men.

Among Melanesian populations, the youth are perhaps the group most subjected to the dissolution of traditional customs (Foster, 1999, 2002). This is why such an outcome is not unexpected. Our data, together with previous results obtained by Sear and Marlowe (2009) and Sorokowski et al. (2012), showed that, among indigenous populations, the “male-taller norm” still exists, but it is far more flexible than in Western cultures. We hypothesize that in adverse environmental conditions such as in the study site of highland areas of Yalimo, short women were not preferred because lower height in humans can result from malnutrition or various infectious diseases (Beard & Blaser, 2002; Martorell et al., 1975) and may, therefore, be a cue to low “quality” of an individual (in terms of health).

Another analyzed attractiveness marker was LBR. Women did not show preferences for any specific male LBR. At the same time, almost 33% of men rated the female image depicting a .475 LBR as the most sexually attractive. The lowest and highest female LBRs were assessed as the least attractive. Such male preferences were similar to those obtained by Sorokowski et al. (2011) in 27 nations. It confirms that large deviations from the population average are usually perceived as less attractive, probably because they are maladaptive (Symons, 1979).

It is worth noting that, both for men and women, contact with Western culture was not a simple explanation of preferences for relatively long legs. In our sample, we observed the opposite mechanism—participants having less contact with Western culture preferred longer legs. As relatively short legs might indicate low health status, early childhood illnesses or malnutrition and lower reproductive capabilities (Bogin & Varela-Silva, 2010), we presume that those factors might have influenced the preferences of Yali who live in harsh environmental conditions.

In our study, we also found that more than 30% of men rated the female image depicting a .60 WHR as the most sexually attractive. Similar to Europeans (Rozmus-Wrzesinska & Pawlowski, 2005), Yali men preferred a rather low WHR. Therefore, our results were inconsistent with a few previous studies conducted among native societies, where a rather high WHR was preferred. Baksossi men in Cameroon preferred a .80 WHR for both short and long-term relationships (Dixson et al., 2007), as did Shiwiar men in the Equadorian Amazon (Sugiyama, 2004). Initial studies among the Hadza found that a .90 WHR was most attractive to men (Marlowe & Wetsman, 2001; Wetsman & Marlowe, 1999). However, as noted earlier, these results may have been due to the use of front-posed stimuli. Indeed, follow up studies using images in profile view found that Hadza men rated the .60 WHR as most attractive (Marlowe, Apicella, & Reed, 2005). These results, our findings, and those of Singh et al. (2010) confirmed that low WHR can be attractive even in non-Western, indigenous cultures. Nevertheless, our results demonstrated that WHR preferences in the Yali tribe are diverse—the highest WHR was rated as the most attractive by 23% of men. The absence of clear preferences for low WHR might be related to many factors (Sugiyama, 2004; Yu & Shepard, 1998). An additional factor, not considered in previous studies, might be low women’s age at first marriage. In the Yali tribe, like in most indigenous groups, girls are typically married very early. Thus, Yali men’s preferences for female sexual attractiveness might be close to the population average for prepubescent girls (Hammer et al., 1991). If such was the case, higher WHR for females would not necessarily be associated with greater age (Bartali et al., 2002) and therefore lower attractiveness, but also with very young women.

It is not clear if WHR preferences in our sample were related to frequency of contact with other cultures (Indonesian/Western). On the one hand, participants living farther from Wamena (who probably had less contact with Western culture), preferred higher WHR; however, the preferences in Piliam village and other, more remote sites did not differ.

An interesting result (however, observed only at the trend level for WHR) was that the number of pigs possessed by participants was related to their preferences for body composition. Male participants owning less pigs preferred higher WHR (i.e., the preferences of the participants possessing the fewest pigs (first quartile): *M* = .73; possessing the most pigs (fourth quartile): *M* = .66) and higher LBR (i.e., the preferences of the participants possessing the fewest pigs (first quartile): *M* = .468; possessing the most pigs (fourth quartile): *M* = .44). In the Yali tribe, number of pigs is a primary marker of wealth (and hence good nutrition). It was shown that accessibility of food resources in a population can be related to preferred body fat level (Anderson, Crawford, Nadeau, & Lindberg, 1992; Jones, 1996). In populations of lower resources, body fat might be a strong indicator that a woman can withstand the demands of pregnancy and lactation (Frisch & McArthur, 1974). Additionally, preferences for WHR might be even more diverse because of socioeconomic status (SES) rather than ethnicity. Data for Britons and a few ethnic groups inhabiting Malaysia demonstrated that men of high SES preferred slimmer women (Swami & Tovee, 2005). Previous studies have also shown that even in Western societies hunger can influence preference for female body weight—hungry male participants preferred women of higher body weight (Nelson & Morison, 2005; Swami & Tovee, 2006). Knowing that silhouettes of higher WHR are commonly perceived as fatter (Rozmus-Wrzesinska & Pawlowski, 2005), we can presume that this is the reason why high WHR might be more attractive to poorer and malnourished Yali with fewer pigs. Preferences for women with longer legs might be explained similarly, as shorter legs are a reliable marker of early childhood illnesses or malnutrition (Bogin & Varela-Silva, 2010). However, this relationship is not fully clear since it was not observed in the case of SDS preferences and women’s assessments of male LBR.

Our study had a few limitations related to stimuli construction. As stated in the procedure, we did not control the WHR of the sample (however, it was not controlled in the majority of such research) and as Yali people are generally very thin (women do not have much adipose tissue in the hips/buttocks area), it is possible that our stimuli did not accurately resemble WHRs in this population. It is also important to note that in some previous studies regarding WHR men had the option to choose from a greater range of WHRs (including higher WHRs than in our study). The highest WHR that could have been chosen by Yali men was .80. Additionally, the pictures used in the SDS study differed from the typical Yali silhouettes (they were not dark-skinned and they seemed fatter than an average Yali). In addition, the construction of the SDS sets, taken from the Pawlowski (2003) study seems to have involved enlarging female silhouettes, not only their elongation. Thus, the tallest women seemed also “larger”—with a little larger breasts, thighs, etc. On the other hand, if the female silhouettes were only elongated, and not enlarged, the tallest silhouettes would seem excessively slim and the shortest excessively fat. However, further research is necessary to explore the influence of stimuli construction on the observed SDS preferences. Another limitation for all our studies is the lack of a concurrent Western comparison group. However, usage of stimuli taken from previous studies on Western populations (WHR: Rozmus-Wrzesinska & Pawlowski, 2005; SDS: Fink et al., 2007; Pawlowski, 2003; LBR: Sorokowski & Pawlowski, 2008, Sorokowski et al., 2011) enables us to compare observed preferences with past results.

In conclusion, our study showed that the mate preferences among Yali men and women for WHR, LBR, and SDS were not exactly the same as in Western samples. Generally, our results regarding all body composition elements support the outcome of studies suggesting that aesthetic preferences are malleable and can change with exposure to different environments and cultures (Tovée, Maisey, Emery, & Cornelissen, 2006; Yu & Shepard, 1998), which possibly allows us to adapt to different ecological conditions.
